# pMAGs: A Versatile and Efficient Vector System for Multi-Gene Studies in Plants

**DOI:** 10.3390/plants14162602

**Published:** 2025-08-21

**Authors:** Mengyue Zhang, Jing Liu, Han Zhao, Zhaojun Ding, Xiaoxuan Li, Zipeng Yu

**Affiliations:** The Key Laboratory of Plant Development and Environmental Adaptation Biology, Ministry of Education, School of Life Sciences, Shandong University, Qingdao 266237, China; mengyuezhang0808@foxmail.com (M.Z.); liujing20020123@163.com (J.L.); zhaohan020204@163.com (H.Z.); dingzhaojun@sdu.edu.cn (Z.D.)

**Keywords:** multi-gene co-expression, pMAG vectors, fluorescence visualization, hairy roots, stable transformation

## Abstract

In molecular biology studies, suitable vectors are fundamental tools; however, most vectors can only express one target gene, which limits the ability to study multiple genes simultaneously within the same plant tissue. The traditional method for achieving multi-gene co-expression involves co-transferring multiple plasmids into plant tissues, but this approach is often inefficient due to the difficulty of successfully transforming multiple plasmids at once. To overcome this limitation, we have developed a series of vectors, called pMAGs (Multigene Assembly Genetic vectors), capable of simultaneously expressing or silencing two or three different genes in plants. These vectors not only provide an optimal solution for a wide range of biological experiments but also work effectively across numerous plant species.

## 1. Introduction

With the advent of the post-genomic era, research has shifted toward studying gene functions [1,2,3,4]. Generating transgenic plants and mutants with functional deficiencies is a crucial strategy for investigating plant gene functions [5,6]. Researchers can utilize various vectors for *Agrobacterium tumefaciens*-mediated transformation to produce desired transgenic plants. For instance, clustered regularly interspaced palindromic repeats (CRISPR)/Cas9 or artificial microRNA vectors can be employed to generate mutants, while overexpression vectors facilitate the production of overexpressed plants. Promoter-driven reporter genes enable analysis of tissue-specific expression patterns of target genes, and fluorescent protein fusions can be used to study the subcellular localization of target proteins [7,8,9,10,11]. Several cloning methods have been developed to insert genes into vectors, including enzymatic ligation, Gateway, Golden Gate, and Gibson Assembly (GA) [12,13,14,15,16]. As the cost of recombinant enzymes decreases, GA has become mainstream due to its low cost and high efficiency. Overall, the development of these cloning techniques has greatly advanced research in the molecular, biochemical, and genetic fields.

Transformation of some crops is difficult and time-consuming, which seriously restricts the study of molecular mechanisms in these crops. Equally, root nodule symbiosis and root disease resistance have long been focal research areas in leguminous plants. Transient expression systems, such as transformation in hairy roots or protoplasts of these crops, serve as effective alternatives [8,17,18]. The hairy root transformation system is widely used as a research tool to investigate root development, the mechanisms underlying nodule symbiosis and the regeneration to form buds [6,17,19,20,21,22]. Nonetheless, the positive hairy root identification typically relies on real-time quantitative polymerase chain reaction (RT-qPCR) or target gene amplification [8,23]. With the use of many fluorescent proteins, the identification of transformed plants is becoming easier [9,20,21,24]. Additionally, in previous transient transformation systems, co-transforming two genes typically involved constructing two separate plasmids, which is much less efficient than cloning both genes into a single vector [11].

Here, we developed pMAG vectors incorporating commonly used tags (MYC, FLAG, and GFP), enabling the co-expression of multiple genes in a single vector. Using pMAG vectors, we found that, by overexpressing GFP, positive roots can be easily identified with a hand-held fluorescent lamp. Moreover, the pMAG vectors are versatile tools for biochemical experiments, including cell-free assays, protein stability analysis, chromatin immunoprecipitation sequencing (ChIP-seq), RNA sequencing (RNA-seq), co-immunoprecipitation (Co-IP), immunoprecipitation-mass spectrometry (IP-MS), subcellular localization study, and analysis of post-translational modifications. They can also be used for hairy root systems, Arabidopsis protoplasts, and tobacco transformation. Thus, the pMAGs system streamlines functional genomics workflows by enabling: (1) rapid gene necessity screening (shortening the decision-making cycle), (2) efficient functional characterization of individual genes, and (3) mechanistic investigation of multi-gene interactions within regulatory pathways—effectively addressing the key stages of screening, characterization, and mechanistic analysis in molecular genetics research.

## 2. Results

**Figure 1:**
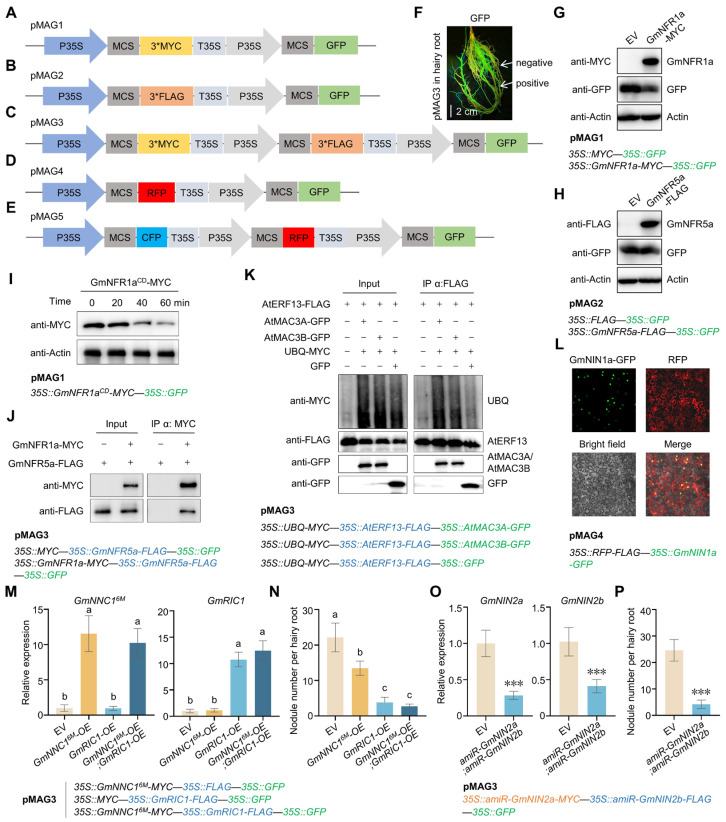
pMAGs with high expression efficiency and wide applicability. (**A**–**E**) Schematic diagrams of pMAGs. (**F**) Fluorescence signals in pMAG3-transformed soybean hairy roots under a hand-held fluorescent lamp. Bar: 2 cm. (**G**) Protein level of GmNFR1a in soybean hairy roots transformed with pMAG1 expressing *GmNFR1a* or empty vector (EV) lacking the *GmNFR1a* gene. (**H**) Protein level of GmNFR5a in soybean hairy roots transformed with pMAG2 expressing *GmNFR5a* or EV lacking the *GmNFR5a* gene. (**I**) Cell-free assay shows the protein levels of GmNFR1a^CD^-MYC in the hairy roots transformed with pMAG1 expressing *GmNFR1a^CD^-MYC*. (**J**) Co-immunoprecipitation (Co-IP) assay detects the interaction of GmNFR1a and GmNFR5a in hairy roots transformed with pMAG3 expressing *GmNFR1a* and *GmNFR5a*. (**K**) Ubiquitination of AtERF13 in the presence or absence of AtMAC3A or AtMAC3B detected in an Arabidopsis protoplast transient transformation assay transformed with pMAG3. (**L**) Protein subcellular localization analysis in *Nicotiana benthamiana* leaves transformed with pMAG4 expressing *RFP* and *GmNIN1a*. (**M**) Transcripts of *GmNNC1^6M^* and *GmRIC1* in soybean hairy roots transformed with pMAG3 expressing *GmNNC1^6M^*, *GmRIC1*, or both. ^a,b^: One-way ANOVA; *p* < 0.05; mean ± SD. (**N**) Nodule number on soybean hairy roots shown in (**M**). ^a–c^: One-way ANOVA; *p* < 0.05; mean ± SD. (**O**) Transcripts of *GmNIN2a* and *GmNIN2b* in hairy roots transformed with pMAG3 expressing *amiR-GmNIN2a* and *amiR-GmNIN2b*. Student’s *t*-test; *** *p* < 0.001; mean ± SD. (**P**) Nodule number on hairy roots shown in (**O**). Student’s *t*-test; *** *p* < 0.001; mean ± SD.

Briefly, we introduced the 35S::MCS-3*MYC and 35S::MCS-3*FLAG fragments into the pSuper1300-GFP framework [25], generating the vectors pMAG1 and pMAG2, respectively (Figure 1A,B). To enable the simultaneous expression of three genes, we further introduced the 35S::MCS-3*MYC fragment into pMAG2 to produce a new vector, pMAG3 (Figure 1C). Co-localization of two proteins is the prerequisite for their interaction. Therefore, we replaced the 3*FLAG tag in pMAG2 with a red fluorescent protein (RFP) tag to obtain a new vector, pMAG4, which can simultaneously express two different fluorescent proteins (Figure 1D). Similarly, we modified the pMAG3 to develop pMAG5, a vector capable of simultaneously expressing three different fluorescent proteins (Figure 1E). Collectively, we have developed a set of vectors with different tag combinations (Figure S1), offering an efficient solution to the challenges of multi-plasmid co-transformation and expanding the versatility of plant molecular biology experiments.

High expression efficiency in plants is essential for vector-wide application in biological research. To evaluate the expression efficiency of pMAGs, we assessed the pMAG-carried genes at both transcriptional and protein levels using the soybean hairy root system. We introduced the nodule development-related gene *Glycine max Rhizobia-Induced CLE1* (*GmRIC1*) or *Glycine max nod factor receptor 1a* (*GmNFR1a*) into pMAG1, *Glycine max nod factor receptor 5a* (*GmNFR5a*) into pMAG2, and *GmNFR1a* and *GmNFR5a* together into pMAG3 (Figure 1F–H and Figure S2). In addition, we expressed *GmNFR5a* alone or *GmNFR5a* and *GmNFR1a* together by pMAG3 (Figure S3). Through the *Agrobacterium rhizogenes*-mediated soybean hairy root transformation system, we demonstrated their effectiveness for plant molecular studies.

The suitability of vectors for various experimental applications is another key indicator of their value; therefore, we tested their performance in multiple protein study methods. The cell-free assay is a well-documented method for assessing protein stability [26]. To evaluate the functionality of pMAGs in this system, we introduced the coding sequence (CDS) of the cytoplasmic domain of GmNFR1a (*GmNFR1a^CD^*) into pMAG1 and detected the protein levels of GmNFR1a^CD^-MYC in the protein extracts from pMAG1-transformed positive hairy roots. The high initial protein quantity of *GmNFR1a^CD^*-MYC and its subsequent regular degradation over time indicate that pMAG1 works efficiently in cell-free systems (Figure 1I). To test pMAGs in co-immunoprecipitation (Co-IP) application, we introduced an interacting protein pair GmNFR1a-GmNFR5a into pMAG3 and detected their interaction in soybean hairy roots [27]. As expected, the Co-IP results revealed a good performance of pMAG3 in detecting protein interactions (Figure 1J). To further evaluate pMAG3 in in vivo ubiquitination experiments, we introduced the ubiquitin ligases (E3) MOS4-ASSOCIATED COMPLEX 3A (AtMAC3A) and AtMAC3B, along with their target ETHYLENE-RESPONSIVE ELEMENT BINDING FACTOR 13 (AtERF13), into pMAG3 and tested its availability in the Arabidopsis protoplast transient transformation system [26]. The results showed that AtMAC3A-GFP and AtMAC3B-GFP, but not GFP alone, enhanced the ubiquitination level of AtERF13, confirming that pMAG3 works efficiently in the protein ubiquitination assays (Figure 1K). In addition, we also evaluated pMAG4 in *N. benthamiana* leaves by introducing the CDS of nucleus-localized *Glycine max Nodule Inception 1a* (*GmNIN1a*) into pMAG4 [28]. We found GmNIN1a-GFP was specifically localized in the nucleus, while RFP was localized in the cytoplasm and nucleus (Figure 1L), confirming that the two different fluorescent proteins in pMAG4 were efficiently expressed in *N. benthamiana* leaves.

Collectively, the pMAG vectors performed well in various biological experiments (Figure 1I–L), demonstrating their versatility across multiple systems. Moreover, their effectiveness in soybean (Figure 1I,J), Arabidopsis (Figure 1K), and *N. benthamiana* (Figure 1L) highlights their broad applicability in different plant species. Nonetheless, all of these experiments are based on transient transformation systems, which raises the unavoidable question: Can pMAGs be continuously expressed in plants for long-term phenotypic analysis? To answer this question, we selected two soybean root nodulation repressors, *Glycine max Nodule Number Control 1* (GmNNC1^6M^; an NNC1 variant that avoids microRNA172c-targeted degradation) and *GmRIC1* [23], which were transferred individually and jointly into pMAG3. After more than 60 days of growth of the pMAG3-transformed hairy roots, we observed that both GmNNC1^6M^ and GmRIC1 significantly repressed root nodule formation (Figure 1M,N), confirming the long-term effectiveness of pMAG3 in plants. Furthermore, through artificial microRNA (amiRNA)-mediated gene silencing, we identified the simultaneous decline of *Glycine max Nodule Inception 2a* (*GmNIN2a*) and *GmNIN2b* transcripts, leading to decreased soybean nodulation in pMAG3-transformed hairy roots (Figure 1O,P). These results confirm that pMAG3 effectively enables multi-gene co-knockdown in plants.

In summary, pMAGs (i) perfectly match various biological experiments; (ii) are widely matched to many plant species; and (iii) are suitable for transient transformation systems and stable transgenic plants generation. Therefore, pMAGs are versatile, efficient, and widely applicable tools that will provide more convenience for scientific research.

## 3. Discussion

Traditionally, the co-expression of multiple genes required cloning each gene into separate vectors, which proved inefficient for co-transforming genes within the same tissue or cell [11]. The pMAG vectors overcome this limitation by incorporating multiple ORFs into a single construct, enabling the simultaneous expression of multiple genes. This approach significantly enhances the efficiency of gene co-expression, making it an ideal system for functional studies involving multiple target genes. For example, the pMAG3 vector allows for co-expression of three genes in hairy roots, facilitating complex experiments such as Co-IP, genetic relationship verification, and protein post-translational modification analyses (Figure 1J,K,M–P). This multi-gene co-expression capability is particularly valuable for studying protein–protein interactions and signaling pathways, as well as modifications such as phosphorylation, ubiquitination, and sumoylation.

For crops with recalcitrant transformation protocols, transient transformation in hairy roots or protoplasts is crucial for rapid phenotypic and molecular analyses. Hairy root transient transformation, for example, has become essential for studying legume–rhizobium interactions [8,17,24,27]. Another key feature of the pMAG vectors is their ability to visualize positive hairy roots by overexpressing GFP. This visual detection system simplifies positive root screening, reducing the need for expensive and specialized fluorescence excitation equipment. Importantly, the GFP tag can be replaced by other visualization proteins such as RUBY protein, allowing positive roots to be easily identified with the naked eye under natural light, and further simplifying the screening process [24].

Coordinated multi-gene expression on single plasmids constitutes a persistent challenge due to unpredictable cis- and trans-regulatory effects of enhancer elements [29,30,31]. In our constructed multigene expression vectors, no significant aberrations in gene expression caused by the presence of multiple promoters were observed across the examined transcriptional profiling (Figure 1M,O and Figure S2), protein levels (Figure 1G–K), or phenotypic analyses (Figure 1N,P).

Potential limitations include the system’s dependency on specific promoters (e.g., CaMV 35S) for broad-host applications. Future work will optimize modular promoters for tissue-specific expression. Additionally, co-expression efficiency in high-copy-number contexts requires further validation in different species. These refinements will enhance adaptability across diverse plant species.

In summary, pMAG vectors represent versatile and efficient tools for functional genomics research. By enabling rapid visualization of positive hairy roots and allowing multi-gene co-expression, these vectors streamline the investigation of gene function, protein–protein interactions, and protein post-translational modifications. Their flexibility and ease of use make them an invaluable resource for both basic research and applied studies in crop biotechnology.

## 4. Materials and Methods

### 4.1. pMAG Vectors Construction

To simultaneously express or silence two or three different genes within the same plant tissues, we have developed a series of vectors called pMAGs (Multigene Assembly Genetic vectors) based on the pSuper1300-GFP vector backbone [25,32]. Briefly, the 35S::MCS-3*MYC and 35S::MCS-3*FLAG fragments were separately amplified and cloned into pSuper1300-GFP by Gibson Assembly (GA) reactions (RK21020, ABclonal, Wuhan, China), producing two vectors, pMAG1 and pMAG2. To enable the simultaneous expression of three genes, we further introduced the 35S::MCS-3*MYC fragment into pMAG2 to create a new vector, pMAG3. Subsequently, we replaced the 3*FLAG tag in pMAG2 with a red fluorescent protein (RFP) tag to obtain a new vector, pMAG4, which can simultaneously express two different fluorescent proteins. Similarly, we modified the pMAG3 to obtain pMAG5, a vector capable of simultaneously expressing three different fluorescent proteins. To facilitate the introduction of interesting genes into pMAG vectors via GA reactions, we inserted multiple cloning sites (MCS) in front of the MYC, FLAG, and GFP tags, respectively. All primers used in the construction of pMAGs are listed in Table S1.

During insertion of target genes, the pMAG vectors are first digested using appropriate restriction enzymes, as shown in Figure S1. The target DNA fragment is then directly assembled into the linearized pMAG vector via GA reactions. When inserting a second gene, the vector containing the first gene is digested again, and the second target fragment is ligated into the vector through another GA reaction. The restriction enzymes selected for the second digestion must not be present within the first inserted gene. Otherwise, the first gene would be cleaved, preventing the formation of a circular plasmid structure. The nucleotide sequences of the MCS and restriction enzyme sites in the pMAG vector series are shown in Figure S1. Suggested enzyme cutting sites and homology arms sequence in the pMAG vector series are shown in Table S2.

### 4.2. Soybean Hairy Root Transformation and Nodulation Phenotypic Analysis

To evaluate the expression efficiency of pMAGs in plants, we introduced pMAGs into soybean hairy roots, and analyzed the expression of pMAGs-carried genes and the development of soybean root nodules. Briefly, the coding sequence (CDS) of *Glycine max nod factor receptor 1a* (*GmNFR1a*) was introduced into *Hind*III-digested pMAG1 vector pre-MYC tag by GA reaction; the CDS of *GmNFR5a* was cloned into *Sal*I-digested pMAG2 vector pre-FLAG tag by GA reaction; the CDS of *GmNFR1a* was cloned into *Hind*III-digested pMAG3 vector pre-MYC tag and the CDS of *GmNFR5a* was cloned into *Sal*I-digested pMAG3 vector pre-FLAG tag by GA reaction; the CDS of *GmNNC1^6M^* (an NNC1 variant that avoids microRNA172c-targeted degradation) was cloned into *Hind*III-digested pMAG3 vector pre-MYC tag, and the CDS of *Glycine max Rhizobia-Induced CLE1* (*GmRIC1*) was cloned into *Sal*I-digested pMAG3 vector pre-FLAG tag by GA reaction; the fragment of *amiR-GmNIN2a* was cloned into *Hind*III-digested pMAG3 vector pre-MYC tag and the fragment of *amiR-GmNIN2b* was cloned into *Sal*I-digested pMAG3 vector pre-FLAG tag by GA reaction. All primers used in these assays are listed in Table S1.

These constructs were transformed into *Agrobacterium rhizogenes* strain K599 with the freeze–thaw method. Positive colonies were transferred into YEB medium (Y003, MDBio, Inc., Qingdao, China; 50 µg mL^−1^ kanamycin) and cultured at 220 rpm for 24 h at 28 °C. For soybean hairy root transformation, Williams 82 (W82) seedlings grown in a 27 °C greenhouse for 4 d were used for *Agrobacterium rhizogenes*-mediated hairy root transformation [8]. Positive hairy root transformants were visualized using a hand-held fluorescent lamp (LUYOR-3415RG, Luyor Instrument, Los Angeles, CA, USA) with filter glasses in the dark. For nodulation phenotypic analysis, the pMAGs-transformed positive soybean seedlings were transferred to the pot (soil:vermiculite = 1:1) and grown in a 12 h light/12 h dark cycle at 27 °C and 60% relative humidity for 2 d. Positive hairy root transformants were then inoculated with *Bradyrhizobium diazoefficiens* USDA110 (OD_600_ = 0.08) suspension once every 7 d for a total of four inoculations. These hairy roots were harvested 28 d after the initial inoculation, and the number of nodules on each hairy root was counted.

### 4.3. RNA Extraction and RT-qPCR

Quantitative real-time polymerase chain reaction (RT-qPCR) assays were performed to determine whether pMAGs can simultaneously express or silence two or three genes within the same plant tissue at the transcriptional level. In brief, approximately 0.5 g of pMAGs-transformed positive hairy roots were collected for RNA extraction and the empty vector-transformed hairy roots were used as the control. Total RNA was extracted using a SPARKeasy rapid Plant RNA extraction kit (AC0305, Sparkjade Biotech Co., Ltd., Jinan, China). About 2 μg of RNA was reverse-transcribed into first-strand complementary DNA (cDNA) with HiScript IV RT SuperMix (R423, Vazyme, Nanjing, China). RT-qPCR was performed on a MyiQ Real-time PCR Detection System (Bio-Rad, Hercules, CA, USA) using the 2× M5 Hiper SYBR Premix Estaq (MF787, Mei5 Biotech, Beijing, China). *GmELF1B* and *GmCYP2* were used as the reference genes. All primers used for RT-qPCR are listed in Table S1.

### 4.4. Western Blot Assay

To verify whether pMAGs can simultaneously express two or three genes within the same plant tissue at the protein level, we collected approximately 1 g of pMAGs-transformed positive hairy roots for protein extraction. In short, the samples were re-suspended in a 300-μL pre-cooled lysis buffer (90% [*v*/*v*] NP40, 1 mM DTT [CD4941, Coolaber, Beijing, China], and 1% [*v*/*v*] Triton X100) that was pre-added with a protease inhibitor cocktail (MB12707-1, Meilunbio, Dalian, China). The GmNFR1a-MYC proteins were detected with an anti-MYC antibody (AE038, ABclonal, 1:5000 dilution, Wuhan, China); the GmNFR5a-FLAG proteins were detected with an anti-FLAG antibody (AE005, ABclonal, 1:5000 dilution, Wuhan, China); the GFP proteins were detected with an anti-GFP antibody (HT801-02, TransGen Biotech, 1:5000 dilution, Beijing, China); and the actin proteins were detected with an anti-actin antibody (K200058M, Solarbio, 1:5000 dilution, Beijing, China). Western blot (WB) images were taken with the Tanon-5200 Multi imaging system (Tanon, Shanghai, China). All primers used for WB assays are listed in Table S1.

### 4.5. Cell-Free Assay

To determine whether pMAG vectors are suitable for cell-free assay, we introduced the CDS of the cytoplasmic domain of GmNFR1a (*GmNFR1a^CD^*) to *Hind*III-digested pMAG1 vector pre-MYC tag by GA reaction and then induced the soybean hairy roots. We detected the protein levels of GmNFR1a^CD^-MYC in the cell-free system, and the cell-free assays were performed as previously described [33]. The GmNFR1a^CD^-MYC proteins were detected with an anti-MYC antibody (AE038, ABclonal, 1:5000 dilution, Wuhan, China), and the actin proteins were detected with an anti-actin antibody (K200058M, Solarbio, 1:5000 dilution, Beijing, China). WB images were taken with the Tanon-5200 Multi imaging system (Tanon, Shanghai, China). All primers used for cell-free assays are listed in Table S1.

### 4.6. Co-IP Assay

To determine whether pMAGs can simultaneously express two proteins within the same plant tissue for the co-immunoprecipitation (Co-IP) experiment, the CDSs of *GmNFR1a* and *GmNFR5a* were cloned into pMAG3 and then the pMAG3-transformed soybean hairy roots were induced. About 1 g of positive hairy roots were collected and immediately ground into powder with liquid nitrogen. The samples were transferred into pre-cooled 10-mL tubes and lysed by 500 µL of pre-cooled lysis buffer (1% [*v*/*v*] Triton X100, 1 mM DTT [CD4941, Coolaber, Beijing, China], protease inhibitor cocktail [MB12707-1, Meilunbio, Dalian, China], and 90% [*v*/*v*] NP40). Then, 50 μM MG132 (A2585, APExBIO, Houston, TX, USA; a 26S proteasome inhibitor) was added to inhibit the protein degradation and the mixture was shaken three times every 10 min. The samples were then centrifuged at 12,000× *g* for 20 min and the supernatant was transferred into a pre-cooled 2-mL tube. Then, 50 μL of supernatant was suctioned as an input, and 20 μL 5× loading buffer (LT101, Epizyme Biotech, Shanghai, China) was immediately added, followed by boiling for 10 min. Anti-MYC magnetic beads (L-1102, 1:100 dilution, Biolinkedin, Shanghai, China) were used for immunoprecipitation of GmNFR1a-MYC. The proteins were denatured by adding 50 µL 2× loading buffer and boiled for 10 min. The GmNFR1a-MYC proteins were detected with an anti-MYC antibody (AE038, ABclonal, 1:5000 dilution, Wuhan, China), and the GmNFR5a-FLAG proteins were detected with an anti-FLAG antibody (K200001M, Solarbio, 1:5000 dilution, Beijing, China). WB images were taken with the Tanon-5200 Multi imaging system (Tanon, Shanghai, China). All primers used for Co-IP are listed in Table S1.

### 4.7. In Vivo Ubiquitination Assay

To determine whether pMAGs can simultaneously express two proteins within the same plant tissue for the in vivo ubiquitination assay, the CDS of *AtUBQ10* was cloned into *Hind*III-digested pMAG3 vector pre-MYC tag, the CDS of *AtERF13* was cloned into *Sal*I-digested pMAG3 vector pre-FLAG tag, and the CDS of *AtMAC3A or AtMAC3B* was cloned into *Kpn*I-digested pMAG3 vector pre-GFP tag. Ubiquitination assays were performed as previously described [26]. The AtUBQ-MYC proteins were detected with an anti-MYC antibody (AE038, ABclonal, 1:5000 dilution, Wuhan, China), the AtERF13-FLAG proteins were detected with an anti-FLAG antibody (K200001M, Solarbio, 1:5000 dilution, Beijing, China), and the AtMAC3A-GFP or AtMAC3B-GFP proteins were detected with an anti-GFP antibody (HT801-02, TransGen Biotech, 1:5000 dilution, Beijing, China). WB images were taken with the Tanon-5200 Multi imaging system (Tanon, Shanghai, China). All primers used for in vivo ubiquitination assay are listed in Table S1.

### 4.8. Agrobacterium Tumefaciens-Mediated Invasion of N. benthamiana Leaves

To determine whether pMAGs can simultaneously express two proteins within the same plant tissue for protein subcellular localization analysis, the CDS of *Glycine max* Nodule Inception 1a (*GmNIN1a*) was amplified and cloned into *Kpn*I-digested pMAG4 vector pre-GFP tag by GA reaction. These constructs were transformed into the *Agrobacterium tumefaciens* strain EHA105 and infiltrated into 26-d-old *N. benthamiana* leaves by syringe-mediated infiltration. The cultivation of *N. benthamiana*, the method of infiltration, and the culture after infiltration were performed as previously described [26,33]. Briefly, the infiltrated *N. benthamiana* was incubated under a normal photoperiod (16 h light/8 h dark, 25 °C, and 60% relative humidity) for 60–72 h. Subsequently, the leaves were collected and observed under a confocal laser-scanning microscope (LSM 880, Zeiss, Oberkochen, Germany). Excitation/emission wavelengths were 488 nm/490–560 nm for GFP and 560 nm/580–650 nm for RFP. The fluorescence images were analyzed with ZEN software (version 2.3; Zeiss). All primers used for these assays are listed in Table S1.

## 5. Statistical Analysis

Statistical analyses was performed using Student’s *t*-test (ns, no significant difference; * *p* < 0.05; ** *p* < 0.01; *** *p* < 0.001) or one-way ANOVA (*p* < 0.05) in GraphPad 8 software. The figures were prepared with Microsoft Office PowerPoint and GraphPad 8.

## Data Availability

Data are contained within the article and Supplementary Materials.

## Supplementary Materials

The following supporting information can be downloaded at: https://www.mdpi.com/article/10.3390/plants14162602/s1. Figure S1. Schematic diagrams of pMAGs. Figure S2. Efficient expression of pMAGs in plants. Figure S3. Protein level of GmNFR5a in single-gene vector and multi-gene vector. Table S1. Primers used in this study. Table S2. Suggested enzyme cutting sites and homology arms sequence in pMAGs.
