# Supplementary material for: pMAGs: A Versatile and Efficient Vector System for Multi-Gene Studies in Plants
Source: Plants (Basel). 2025 Aug 21;14(16):2602. doi: 10.3390/plants14162602 (PMC12389698; doi:10.3390/plants14162602)
Supplement: Supplementary file 1 [file plants-14-02602-s001.zip › plants-3772006-supplementary.pdf]

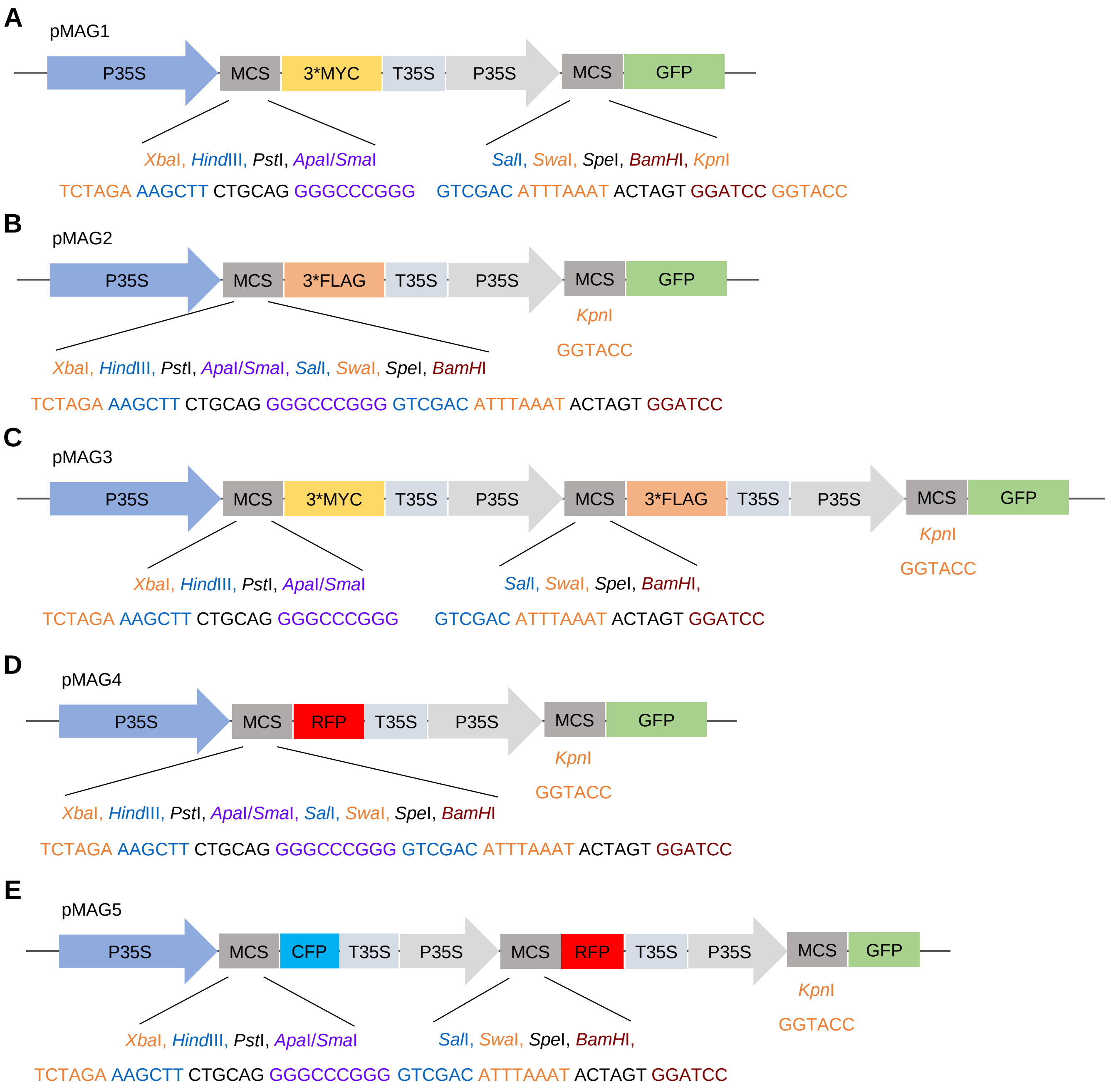

Figure S1. Schematic diagrams of pMAGs.
(**A**–**E**) Multiple clonal sites (MCS) and nucleotide sequences in pMAG1, pMAG2, pMAG3, pMAG4, and pMAG5 vectors.

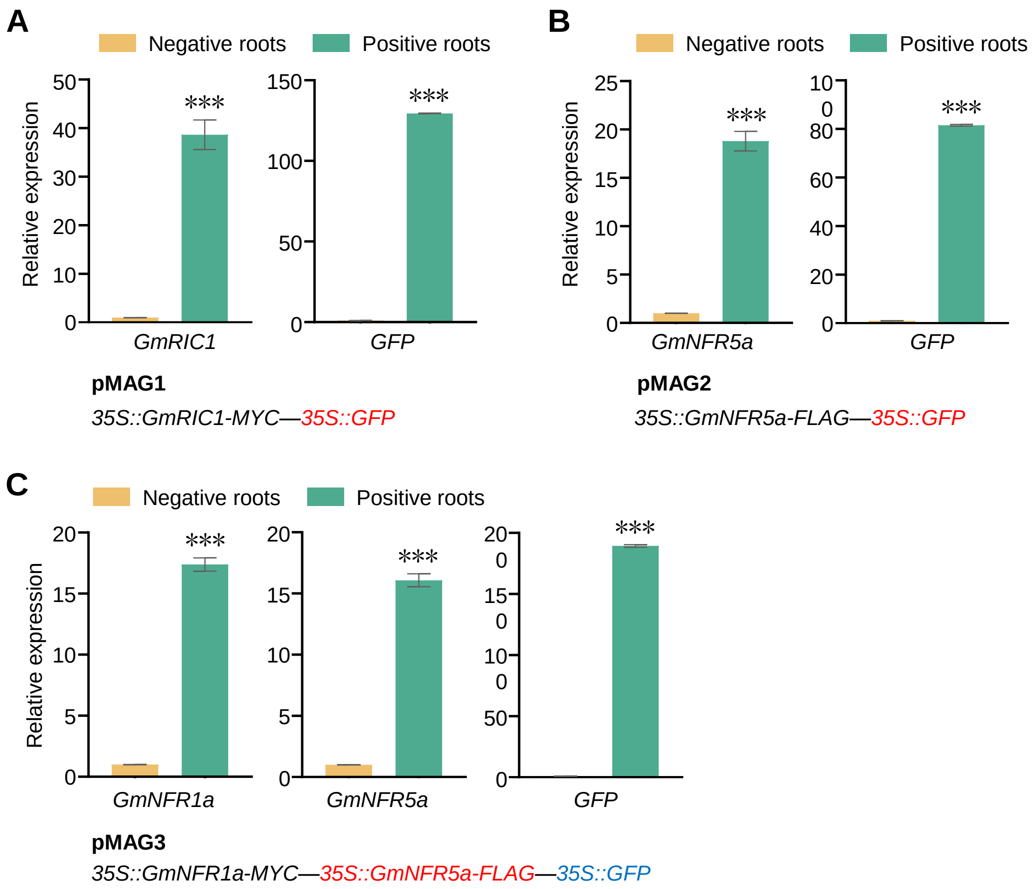

Figure S2. Efficient expression of pMAGs in plants.

(**A**) Expression levels of *GmRIC1* and *GFP* in soybean hairy roots transformed with or without pMAG1 expressing both *GmRIC1* and *GFP*. Student's *t*-test; mean ± SD; ****P* < 0.001. (**B**) Expression levels of *GmNFR5a* and *GFP* in soybean hairy roots transformed with and without pMAG2 expressing both *GmNFR5a* and *GFP*. Student's *t*-test; mean ± SD; ****P* < 0.001. (**C**) Expression levels of *GmNFR1a*, *GmNFR5a*, and *GFP* in soybean hairy roots transformed with or without pMAG3 expressing *GmNFR1a*, *GmNFR5a*, and *GFP*. Student's *t*-test; mean ± SD; ****P* < 0.001.

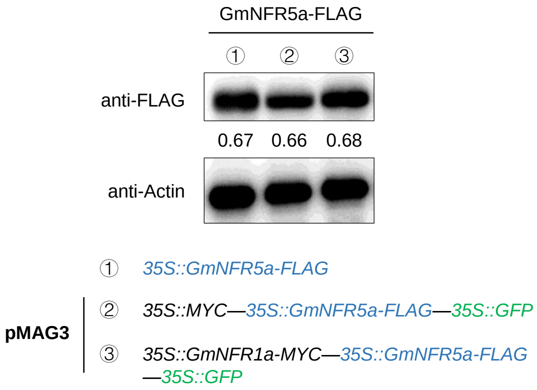

Figure S3. Protein level of GmNFR5a in single-gene vector and multi-gene vector. Protein level of GmNFR5a in soybean hairy roots transformed with single-gene vector, pMAG3 expressing GmNFR5a, and pMAG3 expressing GmNFR5a with GmNFR1a.

Table S1. Primers used in this study.

Purpose	Name	Sequence (5'-3')
pMAG1 construction	GmRIC1-MAG1-F	**ATCGACTCTAGAAAGCTT**ATGGCAAATGCAACAATGGCTAC
	GmRIC1-MAG1-R	**CCCGGGCCCCTGCAG**GTTTCTTGGTGGTGTTGCAAAATTATG
	GmNFR1a-MAG1-F	**ATCGACTCTAGAAAGCTT**ATGGAACTCAAAAAAGGGTTACTTG
	GmNFR1a-MAG1-R	**CCCGGGCCCCTGCAG**TCTCACAGACAGTAGATTTATGAGAG
	GmNFR1a^{CD}-MAG1-F	**ATCGACTCTAGAAAGCTT**ATGAAGTACTTCCAGAAGAAGGAAGG
	GmNFR1a^{CD}-MAG1-R	**ATCGACTCTAGAAAGCTT**TCATCTCACAGACAGTAGATTTATGAGAG
pMAG2 construction	GmNFR5a-MAG2-F	**CTGCAGGGGCCCGGG**ATGGCTGTCTTCTTTCCCTTTCTTC
	GmNFR5a-MAG2-R	**actagtatttaaatg**ACGAGCTGCTATGGAAGTGACAAT
pMAG3 construction	GmNFR1a-MAG3-F	**AAATCGACTCTAGAAAGCTT**ATGGAACTCAAAAAAGGGTTACTTG
	GmNFR1a-MAG3-R	**CCCGGGCCCCTGCAG**TCTCACAGACAGTAGATTTATGAGAG
	GmNFR5a-MAG3-F	**attctacaactacagtcgac**ATGGCTGTCTTCTTTCCCTTTCTTC
	GmNFR5a-MAG3-R	**actagtatttaaatg**ACGAGCTGCTATGGAAGTGACAAT
	AtUBQ10-MAG3-F	**AAATCGACTCTAGAAAGCTT**ATGCAGATCTTTGTTAAGACTCTCAC
	AtUBQ10-MAG3-R	**CCCGGGCCCCTGCAG**ACCACCACGGAGCCTGAG
	AtERF13-MAG3-F	**attctacaactacagtcgac**ATGAGCTCATCTGATTCCG
	AtERF13-MAG3-R	**actagtatttaaatg**TATCCGATTATCAGAATAAGAACA
	AtMAC3A-MAG3-F	**ATTCTACAACTACAGGTACC**ATGAATTGTGCAATTTCCGG
	AtMAC3A-MAG3-R	**GCCCTTGCTCACCAT**TGAATCTTGTGCTGAATCT
	AtMAC3B-MAG3-F	**ATTCTACAACTACAGGTACC**ATGAACTGTGCAATTTCAGG
	AtMAC3B-MAG3-R	**GCCCTTGCTCACCAT**CGAGTCTTGCGCAGAGTCAT
	GmNNC1^{6m}-MAG3-F	**AAATCGACTCTAGAAAGCTT**ATGTTAGATCTTAATCTCAATGCGG
	GmNNC1^{6m}-MAG3-R	**CCCGGGCCCCTGCAG**TGGTGGTGCCTGCGGGGG
	GmRIC1-MAG3-F	**attctacaactacagtcgac**ATGGCAAATGCAACAATGGCTAC
	GmRIC1-MAG3-R	**actagtatttaaatg**GTTTCTTGGTGGTGTTGCAAAATTATG
	amiR-GmNIN2a-MAG3-F	**AAATCGACTCTAGAAAGCTT**CTGCAAGGCGATTAAGTTGGGTAAC
	amiR-GmNIN2a-MAG3-R	**CCCGGGCCCCTGCAG**GCGGATAACAATTTCACACAGGAAACAG
	amiR-GmNIN2b-MAG3-F	**attctacaactacagtcgac**CTGCAAGGCGATTAAGTTGGGTAAC
	amiR-GmNIN2b-MAG3-R	**actagtatttaaatg**GCGGATAACAATTTCACACAGGAAACAG
35S::GmNFR5a-FLAG	*GmNFR5a-FLAG-F*	**CAAATCGACTCTAGA**ATGGCTGTCTTCTTTCCCTTTCTTC
	GmNFR5a-FLAG-R	**actagtatttaaatg**ACGAGCTGCTATGGAAGTGACAAT
pMAG4 construction	RFP-MAG4-F	**cgacatttaaatactagt**Atggcctcctccgaggacg
	RFP-MAG4-R	**TGTAGTCCATggatcc**actctagcatggccgcttaatca
	GmNIN1a-MAG4-F	**tctacaactacagtcgac**ATGGAATATGGTGGGTTGGTGCAG
	GmNIN1a-MAG4-R	**actagtatttaaatg**GGAGGGGCTGGTGCCTCGA
pMAG5 construction	RFP-MAG5-F	**cgacatttaaatactagt**Atggcctcctccgaggacg
	RFP-MAG5-R	**TGTAGTCCATggatcc**actctagcatggccgcttaatca
	CFP-MAG5-F	**TTCTGCAGGGGCCCGGG**ATGGTTTCTAAAGGTGAAGAACTC
	CFP-MAG5-R	**ATGAGCTTTTGCTCCAT**TGCCTTGTAAAGCTCATCCATGC
RT-qPCR	GmRIC1-qPCR-F	CAAATGCAACAATGGCTACTCG
	GmRIC1-qPCR-R	GCCATGGAGATTACTAGCCTGC
	GmNNC1^{6m}-qPCR-F	CAATGGGCAGGAAAGAGC
	GmNNC1^{6m}-qPCR-R	ATGGCAGTCGATGGAAAGGT
	GmNFR1a-qPCR-F	GGTGACATTTCTCCAAAAGT
	GmNFR1a-qPCR-R	GATTAAGTGCTTCTTCAAACAAA
	GmNFR5a-qPCR-F	TTACATGGTGTCAGAGGATAA
	GmNFR5a-qPCR-R	GTGGACTATTCTTGGATAAGC
	GFP-qPCR-F	CGCACCATCTTCTTCAAG
	GFP-qPCR-R	GCCATGATATAGACGTTGTG
	GmNIN2a-qPCR-F	ATCTCAATCTCAATCTCAATCTC
	GmNIN2a-qPCR-R	CCTTGGTTCGTCTCTTCT
	GmNIN2b-qPCR-F	CAACAACTCATCCTCTCCTA
	GmNIN2b-qPCR-R	AGTAGCCTTCACTCTCAAG
	GmELF1B-qPCR-F	GTTGAAAAGCCAGGGGACA
	GmELF1B-qPCR-R	TCTTACCCCTTGAGCGTGG
	GmCYP2-qPCR-F	CGGGACCAGTGTGCTTCTTCA
	GmCYP2-qPCR-R	CCCCTCCACTACAAAGGCTCG

Table S2. Suggested enzyme cutting sites and homology arms sequence in pMAGs.

Vector	Suggested enzyme cutting sites	Homology arms sequence (5'-3')
pMAG1	*Hind*III	ATCGACTCTAGAAAGCTT
		CCCGGGCCCCTGCAG
	*Sal*I	tctacaactacagtcgac
		actagtatttaaatg
pMAG2	*Sal*I	CTGCAGGGGCCCGGG
		actagtatttaaatg
	*Kpn*I	ACATTCTACAACTACA
		CCCTTGCTCACCATGGTACC
pMAG3	*Hind*III	AAATCGACTCTAGAAAGCTT
		CCCGGGCCCCTGCAG
	*Sal*I	attctacaactacagtcgac
		actagtatttaaatg
	*Kpn*I	ACATTCTACAACTACA
		CCCTTGCTCACCATGGTACC
pMAG4	*Sal*I	CTGCAGGGGCCCGGG
		actagtatttaaatg
	*Kpn*I	ACATTCTACAACTACA
		CCCTTGCTCACCATGGTACC
pMAG5	*Hind*III	AAATCGACTCTAGAAAGCTT
		CCCGGGCCCCTGCAG
	*Sal*I	attctacaactacagtcgac
		actagtatttaaatg
	*Kpn*I	ACATTCTACAACTACA
		CCCTTGCTCACCATGGTACC
